# Genetic Diversity and Population Structure: Implications for Conservation of Wild Soybean (*Glycine soja* Sieb. et Zucc) Based on Nuclear and Chloroplast Microsatellite Variation

**DOI:** 10.3390/ijms131012608

**Published:** 2012-10-03

**Authors:** Shuilian He, Yunsheng Wang, Sergei Volis, Dezhu Li, Tingshuang Yi

**Affiliations:** 1 China Southwest Germplasm Bank of Wild Species, The Key Laboratory of Biodiversity and Biogeography, Kunming Institute of Botany, Chinese Academy of Sciences, Kunming 650201, China; E-Mails: heshuilian@mail.kib.ac.cn (S.H.); wys3269@126.com (Y.W.); 2 University of Chinese Academy of Sciences, Beijing 100049, China; 3 College of Horticulture, South China Agricultural University, Guangzhou 510642, China; 4 The Key Laboratory of Biodiversity and Biogeography, Kunming Institute of Botany, Chinese Academy of Sciences, Kunming 650201, China; E-Mail: svolis@gmail.com

**Keywords:** *Glycine soja*, microsatellite, genetic diversity, genetic structure, bottleneck effect

## Abstract

Wild soybean (*Glycine soja* Sieb. et Zucc) is the most important germplasm resource for soybean breeding, and is currently subject to habitat loss, fragmentation and population decline. In order to develop successful conservation strategies, a total of 604 wild soybean accessions from 43 locations sampled across its range in China, Japan and Korea were analyzed using 20 nuclear (nSSRs) and five chloroplast microsatellite markers (cpSSRs) to reveal its genetic diversity and population structure. Relatively high nSSR diversity was found in wild soybean compared with other self-pollinated species, and the region of middle and lower reaches of Yangtze River (MDRY) was revealed to have the highest genetic diversity. However, cpSSRs suggested that Korea is a center of diversity. High genetic differentiation and low gene flow among populations were detected, which is consistent with the predominant self-pollination of wild soybean. Two main clusters were revealed by MCMC structure reconstruction and phylogenetic dendrogram, one formed by a group of populations from northwestern China (NWC) and north China (NC), and the other including northeastern China (NEC), Japan, Korea, MDRY, south China (SC) and southwestern China (SWC). Contrib analyses showed that southwestern China makes the greatest contribution to the total diversity and allelic richness, and is worthy of being given conservation priority.

## 1. Introduction

Soybean [*Glycine max* (L.) Merrill, Fabaceae], is the world’s most important grain legume crop for its protein and oil [1,2], and its genetic diversity has been declining during processes of domestication and artificial selection [2]. Wild soybean (*Glycine soja* Sieb. et Zucc), the ancestor of soybean, retains useful genetic variation for breeding improvement of yield, and resistance to pests, diseases, alkali and salt, and therefore is extremely important germplasm to enrich the soybean gene pool [3].

**Figure 1:**
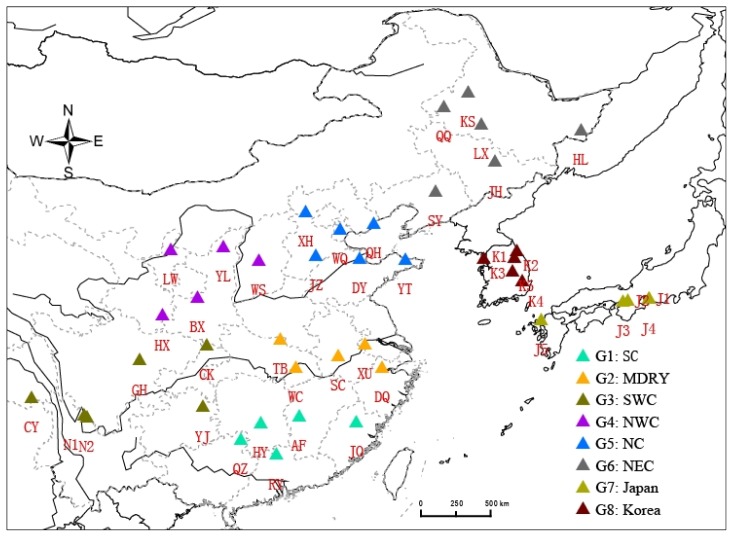
Sampling populations of wild soybean.

Wild soybean is mainly distributed in the Asiatic Floristic region including most of China (53°–24°N and 134°–97°E) [4], the Korean peninsula, the main islands of the Japanese archipelago and Far Eastern Russia [5] (Figure 1). Wild soybean resources have been severely depleted in China in the last 20 years due to habitat fragmentation [6]. Comparing with surveys in 1979 to 1983, the survey conducted by the Chinese Ministry of Agriculture in 2002 to 2004 revealed large range reductions of wild soybean [7]. For example, the most important populations of wild soybean in Jixian county of Heilongjiang province in China have disappeared following land conversion for agriculture; a large population of 0.02 km^2^ in the Keshan county of the same province has been almost completely destroyed, and the large population in the Zhangwu county of the Liaoning province in China has disappeared, leading to the permanent loss of the white-flowered soybean type [7]. Wild soybean has been listed as a national second-class protected plant in 1999 in China [8] and the species requires urgent conservation actions.

The genetic diversity and genetic structure of wild soybean have been studied using morphological traits [3,9], isozymes [10], RFLP [11,12], cytoplasmic DNA [11,13] and SSR markers [14–18]. However, these studies were restricted to particular region(s) and most had a limited sample size [19–22]. These studies produced conflicting results with regards to the diversification of wild soybean. For example, the Korean peninsula [14], northeastern China [3], the Yangtze River region [23], and Southern China [24,25] have all been considered as the center of the species’ diversity by different studies. In order to make appropriate conservation recommendations, a study of the level and geographical structure of the genetic variation across the whole species range is urgently needed. Widely distributed across all eukaryotic genomes, the simple sequence repeat (SSR) is a marker of choice for the analysis of genetic variation [26], with more than 1,000 SSRs markers available for wild soybean [27]. We employed 20 nSSRs and five cpSSRs to study: (i) the extent and structure of genetic variation in wild soybean sampled throughout most of its natural range; and (ii) the demographic history of wild soybean to infer historical changes in population sizes.

## 2. Results

### 2.1. Equilibrium Test and Genetic Diversity

MICROCHECKER found no evidence of scoring errors, but some samples were detected to have null alleles. We failed to amply these alleles despite two to three more genotyping attempts. Mutations in the flanking region may prevent the primer from annealing to template DNA during amplification of microsatellite loci by PCR [28], but we still kept these loci for further analyses because the frequency was relatively small (<5%). All populations deviated significantly from Hardy-Weinberg equilibrium (*p* < 0.05), with the observed heterozygosity being lower than expected (mean observed 0.031, range 0.000–0.205 *vs*. expected 0.426, range 0.018–0.797).

All nSSR loci were polymorphic in all populations. The mean allele richness (*A*_R_) and Shannon’s information index (*I*) were 1.9 (1–3.1) and 0.793 (0.034–1.744), respectively. The fixation index was high (mean 0.913, range 0.202–1). The outcrossing rate was low (mean 8.1%, range 0%–66.4%), but three populations (SY, J2, and K2) showed atypically high outcrossing rate (>48%). The region of MDRY had the highest genetic diversity (*A*_R_ = 14.0, and *I* = 2.349). Observed heterozygosity (*H*_O_ = 0.063) and expected heterozygosity (*H*_E_ = 0.881) of this region were also higher than other regions (Table 1).

All cpSSR loci showed relatively low diversity, the mean allele richness (*A*_R_) and Shannon’s information index (*I*) for cpSSRs were 1.6 (1–3.2) and 0.793 (0–0.908). CpSSRs indicated that Korea has the highest allelic richness (*A*_R_ = 4.6) and Shannon’s information index (*I* = 0.932) among all regions.

**Figure 2:**
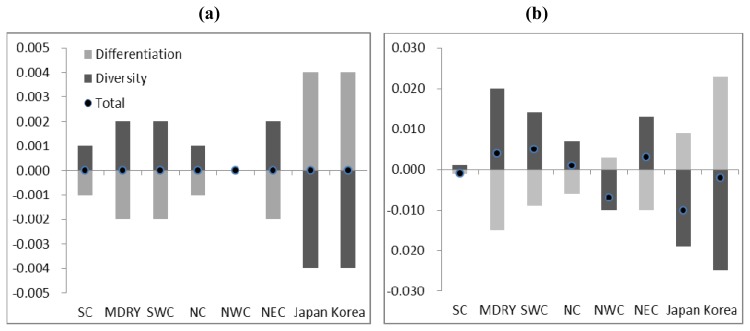

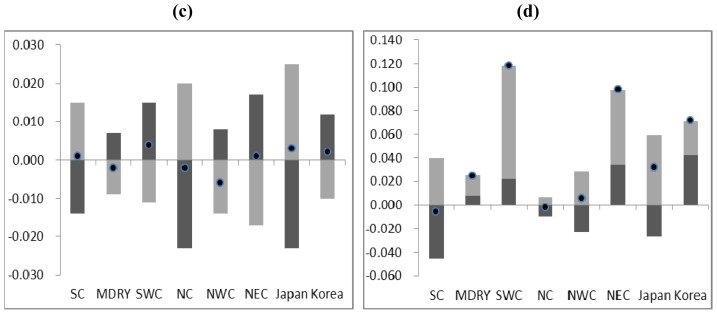
Region contribution to the total diversity and allelic richness, (**a**) and (**b**) for nSSRs; (**c**) and (**d**) for cpSSRs. (**a**) Contribution to total diversity (CT); (**b**) Contribution to allelic richness (CTR); (**c**) Contribution to total diversity (CT); (**d**) Contribution to allelic richness (CTR).

For nSSRs, CONTRIB revealed no difference in regional contribution to total diversity. However, for allelic richness, the highest contribution was made by the SWC region, followed by the regions MDRY and NEC, mainly due to their high own diversity. The lowest contributions came from regions NWC and Japan. For cpSSRs, the SWC region made the greatest contribution to total diversity and allelic richness due to both diversity and differentiation. Besides, the regions of NEC, Korea and Japan made high contributions to allelic richness due to differentiation (Figure 2).

### 2.2. Population Structure

**Figure 3:**
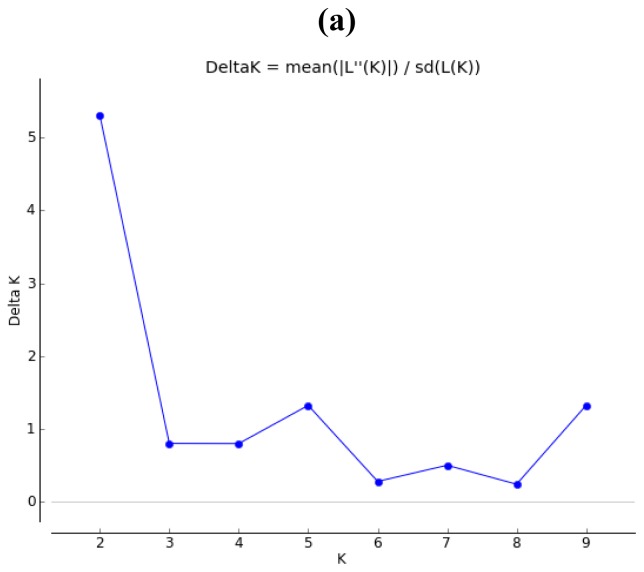

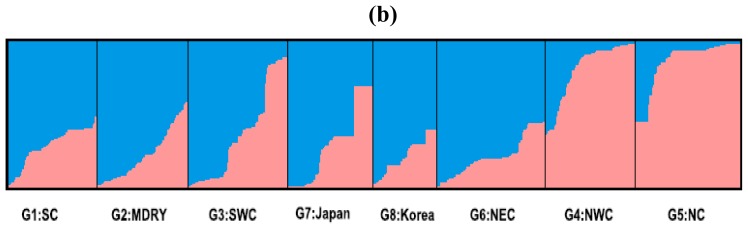
Inferred population structure based on 604 samples and 20 nSSRs. (**a**) DeltaK from STRUCTURE; (**b**) Genetic structure of wild soybean inferred from the admixture model.

**Figure 4:**
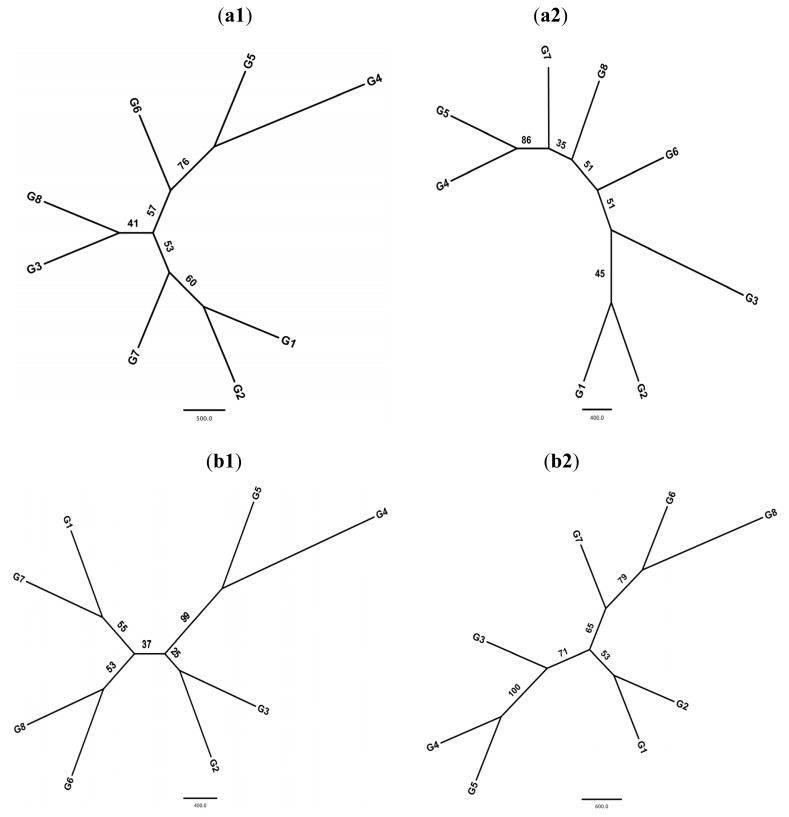
The UPGMA tree of wild soybeans from different regions. bootstrap values are indicated at each branch. (**a**) The cpSSR tree basing on eight groups. **a1**: UPGMA tree; **a2**: Neighbor-joining tree; (**b**) The nSSR tree basing on eight groups. **b1**: UPGMA tree; **b2**: Neighbor-joining tree.

MCMC structure reconstruction of nSSRs showed moderate genetic structure. When Evanno’s [29] *ad hoc* estimator of the actual number of clusters was used, ΔK indicated modes at *K* = 2 (Figure 3a). The average percentages of membership for eight geographical regions of individuals in each of the two clusters were calculated. Most samples (>66%) of the group SC, MDRY, SWC, NEC, Japan and Korea were assigned to cluster 1, and most individuals of group NEC (79.4%) and NC (86.5%) to cluster 2 (Table 2, Figure 3b). No geographic structure was detected for cpSSRs. The UPGMA dendrogram of both nSSRs and cpSSRs divided the eight regions into the same two geographical clusters (Figure 4).

Analysis of nSSRs by AMOVA revealed that 6.0% of genetic variation was due to the genetic distance between the two clusters, 46.7% among populations within clusters and 47.3% between individuals within populations. Similar results were obtained from cpSSRs (6.8%, 57.0% and 36.25%, respectively) (Table 3). A mantel test indicated a significant isolation by distance for cpSSRs (*r*^2^ = 0.021, *p* = 0.002), but not for nSSRs (*r*^2^ = 0.004, *p* = 0.074).

The allele size permutation test rendered non-significant differences between *F*_ST_ and *R*_ST_ estimates (*p* = 0.004 for nSSRs and *p* = 0.01 for cpSSRs; 10,000 iterations), indicating *R*_ST_ estimates were more appropriate than *R*_ST_ for our data. We found high population genetic differentiation (*R*_ST_) (cpSSRs: 0.499 and nSSRs: 0.622). For cpSSRs, the overall level of inferred gene flow (*Nm*) was 0.502 individuals per generation among the populations; and for nSSRs, the gene flow (*Nm*) was 0.251.

### 2.3. Demographic History

Standardized differences test and Wilcoxon sign-rank test based on both SMM and TPM model showed recent reduction in seven populations: Chengkou (CK), Wuchang (WC), Tongbai (TB), Keshan (KS), Jizhou (JZ), Japan1 (J1) and Korea5 (K5). A recent bottleneck effect was also detected in three additional populations of Wuqing (WQ), Jiaohe (JH) and Japan 5 (J5) using TPM model by the Wilcoxon sign-rank test (Table 4). The mode-shift test in allele frequency attributed L-shaped distribution to all populations, which was consistent with normal frequency class distribution ranges (*p* > 0.05).

## 3. Discussion

### 3.1. Genetic Diversity in Wild Soybean

The genetic diversity of wild soybean was studied previously using SSRs [21,25,26,30,31]. However, this is the first time a study uses both nuclear and plastid SSRs to analyze the extent and structure of genetic variation across the whole species range. Wild soybean showed a relatively high population diversity (*H*_E_ = 0.426), which is similar to the result from previous studies [31,32], Considering life form and breeding system have a highly significant influence on genetic diversity [33], we compared genetic diversity of wild soybean with other predominantly self-pollinated wild species, such as wild emmer (*Triticum turgidum* ssp. *dicoccoides*) (*H*_E_ = 0.19) [34], wild barley (*Hordeum spontaneum*) (*H*_E_ = 0.138) [35], and officinal wild rice (*Oryza officinalis*) (*H*_E_ = 0.22) [36]. This may be caused by the special seed dispersion of the wild soybean, the pod dehiscence could discharge the mature seeds to a distance of 0–5 m (up to 6.5 m) [22]. High outcrossing rate for certain populations maybe another reason for high genetic diversity in wild soybean.

The nSSRs showed that MDRY region has the highest diversity, which is consistent with several previous studies. For example, Shimamoto *et al*. [13] reported the highest diversity in the Yangtze River region using RFLP markers. Southern China (including regions of MDRY, SWC and SC) was proposed as the wild soybean center of genetic diversity in a study by Wen *et al*. [37] using a combination of SSRs and morphological traits. The same region was also pointed as origin and center of diversity using SSR markers and nucleotide sequences in a study by Guo *et al*. [25]. Compared with previous results, our study applied more detailed regional division, and the center of diversity was similar, but not as obviously different than in previous studies.

Compared with nSSRs (*A*_R_ = 1.9; *I* = 1.794), the cpSSRs showed less diversity (*A*_R_ = 1.6; *I* = 0.932), which is congruent with Powell *et al*. [38], who used both nSSRs and cpSSRs of wild soybean samples from a germplasm bank. Similar results have been observed in other studies using both types of SSR markers in other species [39–41]. This is consistent with low substitution rate of plant chloroplast cpDNA sequences compared with nDNA [42]. The cpSSRs could offer unique insights into ecological and evolutionary processes in wild plant species in some situation [43], Differing from that of nSSRs, cpSSRs revealed that Korea has the highest wild soybean genetic variation.

### 3.2. Genetic Structure of Wild Soybean

Breeding system, life form, effective population size, genetic drift and gene flow are the major evolutionary effects on population genetic structure, with the effect of breeding system being the predominant one [44,45]. Populations of self-fertilizing species are expected to have lower allelic diversity, lower levels of heterozygosity, and high differentiation among populations than populations from outbreeding species [45]. Here, both nSSRs and cpSSRs showed high inter-population genetic differentiation and low gene flow, as expected in the predominantly selfing wild soybean, combined with low seed and pollen dispersal ability. The seed dispersal distance of wild soybean is short, and 95%, 99%, and 99.9% of the produced seeds disperse within 3.5, 5.0, and 6.5 m, respectively after natural pod dehiscence [22], and nearly 81.4% of the loci were found to be positively correlated in the first two distance classes (0–10 m) [6]. Low pollen dispersal ability can be surmised from the estimates of outcrossing rate in wild soybean, which varied from 2.3% (range 2.4%–3.0%) [46] to 13% (range 9.3%–19%) [47] using allozymes and 3.4% (range 0%–37.4%) applying nSSRs [21]. We found a higher mean outcrossing rate (8.1%), with extremely high values in some populations (G5_YT: 21.1%; G6_SY: 66.4%; G7_J2:52.7%; G8_K2: 48.7%). Despite high selfing rates, occasional outcrossing rate can be subsequent. Occasional high outcrossing was detected in other predominant self-pollinated species such as wild barley (*t* = 25.1%) [48]. The high outcrossing rate in some populations of a predominantly selfing species can be a consequence of rare or sporadically occurring specific environmental conditions (temperature, humidity, wind, insect pollination, *etc*.) [48]. In this study, the populations with high outcrossing rate were found in different habitats from all eight eco-regions, and could not be ascribed to a particular abiotic environmental factor, which can suggest an importance of some biotic factor such as high pollinator visiting activity [6], more studies should be carried out to fully resolve this issue.

The UPGMA and Neighbor-joining dendrogram based on Nei’s genetic distance and assignment test revealed two clusters of wild soybean in both nSSRs and cpSSRs. One cluster was formed by the NC and NWC regions, and the other one was formed by six geographic regions including NEC, SWC, SC, MDRY, Korea and Japan. The absence of differentiation among East China, Southern Japan and the Korean Peninsula (CJK region) is surprising. Fluctuations in sea level among the CJK region throughout the Quaternary (or even in the mid-late Tertiary) provided abundant opportunities for population fragmentation and allopatric speciation at the CJK region. Applying nDNA and cpDNA sequences, the previous phylogeographic studies on *Croomia japonica* [49], *Kirengeshoma palmata* [50], and *Platycrater arguta* [51] suggested deep allopatric-vicariant differentiation of disjunct lineages in the CJK region [52]. Wild soybean might have seen a continuous distribution throughout the CJK region through the exposed East China Sea (ECS) basin when the sea level fell by 85-130/140 meters during Last Glacial Maximum (LGM; 24,000–18,000 years before present) [53,54], the disjunct distribution among this region formed following the submergence of ECS land bridge, and there may be insufficient time for lineage sorting and differentiation. Wild soybean has salt resistance [55], and could grow easily in the salty conditions of a sea shore hence they have more chance to migrate along the land bridge among the CJK regions during glacial periods. We could not totally exclude the possibility of exchange of wild soybean among the CJK region *via* long distance dispersal due to disappear of the ECS land bridge. However, it is just a speculation and will need further studies.

### 3.3. Conservation Implications

In this study, a bottleneck effect was detected in seven populations: Chengkou (CK), Wuchang (WC), Tongbai (TB), Keshan (KS), Jizhou (JZ), Japan 1 (J1) and Korea 5 (K5). The CK and JZ populations are from undisturbed habitats with very small population sizes, while another five populations are situated in disturbed habitats: populations WC, J1 and K5 are along roadsides; population AF is beside an abandoned railway; populations KS and TB are along the ridge of some fields. Population KS is a relic from a larger population predating farming reclamation, and only limited individuals are left. In brief, the five populations were significantly affected by anthropological activities. Wild soybean can adapt to a wide variety of habitats with adequate water. However population size of wild soybean will rapidly decrease in the habitat, with subsequent degradation of genetic diversity and allelic richness. Conversation of wild soybean is therefore a priority, and should focus on regions already affected genetically.

When selecting conservation sites one must also consider a population’s contribution to total diversity and allelic richness. The SWC region was inferred to have greatest contribution to total diversity and allelic richness with both nSSRs and cpSSRs. Wild soybean in this region shows an unusually small population size, combined with a fragmented distribution: several populations from Ninglang county of Yunnan province and Chayu county of Xizang province are separated from the main populations by as much as 400 km. Furthermore, both previous *ex situ* and *in situ* conservation initiatives have paid little attention to this region, and only dozens (from a total of 6172) of wild soybean seed accessions from this region have been collected and stored in the Chinese Crop Germplasm Resources databank (http://icgr.caas.net.cn/cgrisintroduction.html). This region deserves high conservation priority.

## 4. Experimental Section

### 4.1. Samples Collection, DNA Extraction and Microsatellite Genotyping

A total of 604 wild soybean individual leaf samples were obtained from 43 populations across most of the species distribution (Figure 1). Five populations represented two countries, Korea and Japan, and 5 to 6 populations represented each of six regions of China (Table 5). Total genomic DNA was extracted from silica gel-dried leaves using the CTAB method of Doyle and Doyle [56]. The extracted DNA was resuspended in 0.1× TE buffer (10 mmol/L Tris-HCl, PH 8.0, 1 mmol/L EDTA) to a final concentration of 50–100 ng/μL.

Genotyping was performed using 20 nSSRs representing all 20 wild soybean linkage groups corresponding to the 20 chromosomes, and five cpSSRs from intergenic regions. All the 25 loci are polymorphic and have been used in previous studies [15,21,57] (Table 6). PCR reactions were performed in 15 μL reactions containing 30–50 ng genomic DNA, 0.6 μM of each primer, 7.5 μL 2× Taq PCR MasterMix (Tiangen Biotech, Beijing, China). PCR amplifications were conducted under the following conditions: 94 °C for 2 min; 35 cycles at 94 °C for 30 s, 50 °C for 40 s, and 72 °C for 1 min; followed by a final extension step at 72 °C for 7 min. Primers are shown in Table 6. All the SSR markers were polymorphic based on electrophoresis performed on an ABI 3730 DNA sequencer (Applied Biosystems, Foster City, CA, USA). Fragment length sizes were scored automatically using the program GeneMapper (Applied Biosystems).

### 4.2. Microsatellite Validation and Diversity

Microsatellite data from each population was tested for amplification errors and null alleles, large allele dropout or stuttering using 1000 randomizations in MICROCHECKER v.2.2.3 [58]. Genepop v. 3.4 online [59] was used to check for deviation from Hardy-Weinberg expectations and between loci in each population using exact tests with 10,000 dememorizations, 100 batches and 1000 iterations. Significance level was adjusted using the sequential Bonferroni correction for multiple comparisons [60]. For nSSRs, the number of alleles per locus (*A*), the numbers of different alleles (*N_a_*), the observed heterozygosities (*H*_O_), expected heterozygosities (*H*_E_), fixation index (*F*_IS_) and Shannon’s information index (*I*) were calculated using GenALEx v. 6.4 [61]; allelic richness (*A*_R_) was calculated by FSTAT 2.9.3.2 [62]; outcrossing rate (*t*) was calculated from the fixation index using the equation *t* = (1 − *F*_IS_)/(1 + *F*_IS_) [63]. For cpSSR, the number of alleles per locus (*A*), the numbers of different alleles (*Na*) and Shannon’s information index (*I*) were calculated using GenALEx v. 6.4 [61]. In each individual, genetic variants at all cpSSR and nSSR sites were combined into haplotypes. Then, each region was characterized for its plastid DNA diversity using the number of haplotypes detected and gene diversity estimated using the program CONTRIB [64]. Contribution of each region to total diversity (*CT*) and to total allelic richness (*CTR*) were calculated according to Petit *et al*. [65].

### 4.3. Population Spatial Structure

Genetic differentiation was investigated using the model based clustering method STRUCTURE 2.1 [66,67] for nSSRs. Burn-in time and replication number were set to 100,000 and 100,000 (further generation following the burn in) for each run, respectively. The number of populations (K) in the model was systematically varied from 1 to 10. In order to decrease the margin of error, the average value of 20 simulations performed for each K was used. We used the Δ*K* method [29] representing the highest median likelihood values to assign wild soybean accessions using the online tool Structure Harvester [68]. For the chosen K value, the run that had the highest likelihood estimate was adopted to assign individuals to clusters. The 10 runs with the lowest DI values for the selected K-value were retained, and their admixture estimates were averaged using CLUMPP v. 1.1.1 [69], applying the greedy algorithm with random input order and 1,000 permutations to align the runs and calculate G’ statistics. Results were visualized using DISTRUCT 1.1 [70].

Nei’s genetic distance (*D*) and Goldstein’s distance [(δμ)^2^] are commonly used for microsatellite. Considered Goldstein’s distance (δμ)^2^ showed bias at small sample sizes and the bias was directly related to the number of alleles and range in allele size [71], a dendrogram based on Nei’s (1978) [72] genetic distance (*D*) between groups was constructed using the UPGMA method implemented in the PHYLIP v. 3.68 [73]. In order to make sure the results of UPGMA method, a neighbor joining tree also was constructed using PHYLIP v. 3.68. A hierarchical analysis of molecular variance (AMOVA) [74] implemented in Arlequin v. 3.11 [75] was used to partition the observed genetic variation into among clusters, among populations within a cluster and among individuals within a population.

Two commonly estimators of population differentiation are *F*_ST_, based on allele identity, and *R*_ST_, which incorporates microsatellite-specific mutation models. We used the allele size permutation test in SPAGeDI [76] to test whether allele sizes were informative in wild soybean microsatellite data set, which would indicate that mutatioin has contributed to differentiation. Because *R*_ST_ was shown to be most appropriate for our data, (see results), the global *R*_ST_ across all samples was calculated in Arlequin v. 3.11 [75]. Gene flow were quantified using the approach of transform estimates of *R*_ST_ into indirect estimates of the average number of migrants exchanged per generation among populations (*Nm*) [77]. Gene differentiation (*R*_ST_) was calculated using AMOVA analyses based on population levels, gene flow was estimated from *R*_ST_ (nSSRs: *Nm =* 0.25(1 − *R*_ST_)/*R*_ST_; cpSSRs: *Nm =* 0.5(1 − *R*_ST_)/*R*_ST_).

### 4.4. Demographic History

We assessed demographic history based on microsatellite data using different and complementary methods. Heterozygosity excess test [78] and mode-shift test [79] from BOTTLENECK 1.2.02 [80] were used to detect the recent population bottleneck. This program conducts tests for recent (within the past 2*N_e_* to 4*N_e_* generations) population bottlenecks that severely reduce effective population size (*N_e_*) and produce an excess in heterozygosity. Heterozygosity excess test was performed under two mutation models: stepwise mutation model (SMM) and two-phase mutation model (TPM). The model of TPM include both 95% single-step mutations and 5% multiple-step mutations, as recommended by Piry [80]. Heterozygosity excess was detected using the one-tailed Wilcoxon sigh-rank test and standardized differences test on 20 nSSR loci [80]. Significance was determined also by the standardized differences and Wilcoxon tests. Mode-shift test detects allele frequency to investigate whether allele frequency distort from the expected L-shaped distribution. During a bottleneck, the loss of rare alleles occurs more rapidly than the associated decrease in expected heterozygosity, as rare alleles do not contribute to *H*_E_ as much as common alleles, and thus distort the allele frequency distribution from its expected L-shaped distribution [78].

## 5. Conclusions

In summary, our results show a relatively high level of genetic diversity and genetic differentiation in wild soybean. Two major genetic clusters were revealed by both structure and phylogenetic reconstruction. The MDRY and Korea regions contain the highest genetic diversity, and SWC contributes the most to total diversity and allelic richness. Significant genetic bottlenecks have affected five populations with obvious human disturbance. Based on these results, conversation of wild soybean should reduce habitat loss by human interference, and the SWC region should be conserved with priority.

## Figures and Tables

**Table 1:** Genetic diversity parameters estimated at 20 nSSRs and 5 cpSSRs in 43 populations of wild soybean.

	nSSRs									cpSSRs			
		
Pops	*N*	*A*	*N_a_*	*A*_R_	*I*	*H_O_*	*H_E_*	*F_IS_*	*t* (%)	*A*	*N_a_*	*A*_R_	*I*
G1:SC	73	199	10	9.5	1.886	0.013	0.809	0.984	0.8	11	2	2.2	0.421
G1_AF	15	64	3	1.9	0.731	0.014	0.397	0.973	1.4	6	1	1.2	0.079
G1_HY	15	88	4	2.4	1.177	0.014	0.605	0.979	1.1	9	2	1.8	0.493
G1_JO	15	45	2	1.6	0.505	0.007	0.300	0.927	3.8	6	1	1.2	0.079
G1_QZ	13	56	3	1.9	0.751	0.015	0.456	0.969	1.6	6	1	1.2	0.133
G1_RY	15	36	2	1.2	0.190	0.018	0.094	0.736	15.2	7	1	1.3	0.098
G2:MDRY	75	292	15	14.0	2.349	0.063	0.881	0.929	3.7	16	3	3.1	0.742
G2_DQ	14	57	3	2.0	0.792	0.051	0.468	0.904	5.0	10	2	2.0	0.502
G2_SC	15	114	6	2.8	1.493	0.023	0.722	0.969	1.6	10	2	1.9	0.369
G2_TB	15	136	7	3.1	1.744	0.007	0.797	0.992	0.4	14	3	2.7	0.809
G2_WC	14	124	6	3.0	1.616	0.053	0.762	0.933	3.5	11	2	2.1	0.578
G2_XU	15	114	6	2.7	1.436	0.205	0.708	0.718	16.4	10	2	1.9	0.402
G3:SWC	83	199	10	9.3	1.884	0.035	0.803	0.956	2.2	18	4	3.5	0.707
G3_CK	15	57	3	2.0	0.827	0.037	0.491	0.934	3.4	7	1	1.3	0.149
G3_CY	12	38	2	1.4	0.343	0.013	0.200	0.957	2.2	7	1	1.4	0.170
G3_GH	14	62	3	1.9	0.761	0.048	0.426	0.759	13.7	7	1	1.4	0.217
G3_N1	12	59	3	1.8	0.691	0.027	0.383	0.898	5.4	12	2	2.2	0.511
G3_N2	11	52	3	1.8	0.647	0.067	0.407	0.761	13.6	9	2	1.8	0.375
G3_YJ	14	51	3	1.8	0.663	0.014	0.414	0.914	4.5	6	1	1.2	0.130
G4:NWC	75	222	11	10.3	1.740	0.026	0.719	0.964	1.8	13	3	2.6	0.447
G4_BX	15	98	5	2.4	1.162	0.028	0.570	0.962	1.9	6	2	2.0	0.487
G4_HX	14	97	5	2.5	1.196	0.071	0.598	0.835	9.0	11	2	2.1	0.475
G4_LW	15	40	2	1.5	0.428	0.007	0.265	0.978	1.1	11	1	1.0	0.000
G4_WS	15	76	4	2.2	0.974	0.017	0.520	0.968	1.6	5	2	1.7	0.342
G4_YL	15	48	2	1.6	0.517	0.010	0.302	0.905	5.0	9	2	1.6	0.284
G5:NC	86	169	8	8.1	1.633	0.009	0.730	0.989	0.6	11	2	2.2	0.598
G5_DY	15	68	3	2.2	0.934	0.018	0.526	0.973	1.4	9	2	2.0	0.518
G5_JZ	15	44	2	1.9	0.674	0.018	0.455	0.930	3.6	10	1	1.4	0.235
G5_QH	15	49	2	1.4	0.403	0.010	0.212	0.975	1.3	7	1	1.4	0.157
G5_WQ	15	81	4	2.3	1.072	0.003	0.555	0.995	0.3	7	2	1.8	0.500
G5_XH	15	48	2	1.7	0.603	0.000	0.367	1.000	0.0	9	2	1.5	0.276
G5_YT	11	23	1	1.1	0.059	0.005	0.037	0.651	21.1	8	1	1.0	0.000
G6:NEC	89	232	12	10.7	1.932	0.035	0.802	0.956	2.2	17	3	3.4	0.878
G6_HL	15	101	5	2.6	1.303	0.037	0.658	0.946	2.8	5	2	1.8	0.411
G6_JH	15	108	5	2.7	1.405	0.082	0.694	0.882	6.3	9	2	2.0	0.434
G6_KS	15	61	3	2.3	0.979	0.003	0.582	0.995	0.3	11	2	1.8	0.474
G6_LX	15	68	3	2.2	0.930	0.028	0.520	0.958	2.2	9	2	2.1	0.571
G6_QQ	15	78	4	2.1	0.946	0.003	0.504	0.995	0.3	11	2	1.7	0.283
G6_SY	14	26	1	1.1	0.118	0.061	0.076	0.202	66.4	9	1	1.2	0.120
G7:Japan	70	157	8	7.6	1.651	0.023	0.759	0.969	1.6	10	2	2.0	0.372
G7_J1	15	63	3	2.2	0.978	0.021	0.563	0.964	1.8	23	2	1.6	0.343
G7_J2	15	23	1	1.0	0.034	0.007	0.018	0.310	52.7	8	1	1.0	0.000
G7_J3	15	24	1	1.1	0.100	0.003	0.064	0.957	2.2	5	1	1.0	0.000
G7_J4	15	64	3	2.0	0.812	0.034	0.467	0.936	3.3	5	1	1.0	0.000
G7_J5	10	57	3	2.0	0.773	0.069	0.450	0.861	7.5	5	1	1.2	0.065
G8:Korea	53	204	10	10.0	1.774	0.039	0.770	0.949	2.6	23	5	4.6	0.932
G8_K1	10	30	2	1.2	0.163	0.000	0.090	1.000	0.0	5	1	1.0	0.000
G8_K2	12	25	1	1.0	0.049	0.013	0.023	0.345	48.7	5	1	1.0	0.000
G8_K3	10	34	2	1.3	0.259	0.000	0.153	1.000	0.0	5	1	1.0	0.000
G8_K4	12	125	6	2.8	1.513	0.092	0.708	0.872	6.8	16	3	2.9	0.776
G8_K5	7	90	5	2.8	1.360	0.100	0.711	0.860	7.5	16	3	3.2	0.908
Mean	14	65	3	1.9	0.793	0.031	0.426	0.913	8.1	9	2	1.6	0.297

*N*: number of samples; *A*: number of alleles; *A*_R_: allele richness; *N_a_*: number of different alleles; *I*: Shannon’s information index; *H_E_*: expected heterozygosity; *H_O_*: observed heterozygosity; *F_IS_*: fixation index; *t*: outcrossing rate.

**Table 2:** Inferred population structure based on 604 samples and 20 nSSRs.

Regions	No. of sample	Cluster 1	Cluster 2
G1: SC	73	0.729	0.271
G2: MDRY	75	0.771	0.229
G3: SWC	83	0.669	0.331
G4: NWC	75	0.206	0.794
G5: NC	86	0.135	0.865
G6: NEC	89	0.812	0.188
G7: Japan	70	0.694	0.307
G8: Korea	53	0.772	0.228

**Table 3:** Analysis of molecular variance (AMOVA) for wild soybean.

Loci	Source of variation	SS	VC	PV (%)	Fixation indices
nSSR	Among two clusters	393.04	0.565	5.99	*F*_CT_ = 0.060
	Among populations within clusters	5106.23	4.409	46.69	*F*_ST_ = 0.527
	Within populations	5050.64	4.469	47.32	*F*_SC_ = 0.497
cpSSR	Among two clusters	68.562	0.095	6.77	*F*_CT_ = 0.068
	Among populations within clusters	935.881	0.802	56.98	*F*_ST_ = 0.637
	Within populations	589.753	0.510	36.25	*F*_SC_ = 0.611

**Table 4:** Results from bottleneck tests of nSSRs: Significance of both tests is indicated in bold.

Populations.	Standardized difference test				Wilcoxm sign test	

	TPM		SMM		TPM	SMM

	T2	*P*	T2	*P*	*P*	*P*
G1_AF	−2.655	0.0040	−3.014	0.0013	0.9914	0.9959
G1_HY	0.713	0.2381	0.307	0.3794	0.1387	0.3108
G1_JO	−0.121	0.4519	−0.303	0.3809	0.5699	0.6282
G1_QZ	0.434	0.3322	0.125	0.4504	0.0715	0.2046
G1_RY	−4.223	0.0000	−4.457	0.0000	1.0000	1.0000
G2_DQ	1.498	0.0670	1.288	0.0989	0.0521	0.0668
G2_SC	0.967	0.1668	0.420	0.3374	0.1081	0.2729
G2_TB	**3.360**	**0.0004**	**2.991**	**0.0014**	**0.0004**	**0.0004**
G2_WC	**2.651**	**0.0040**	**2.235**	**0.0127**	**0.0007**	**0.0018**
G2_XU	0.854	0.1965	0.308	0.3791	0.1841	0.4492
G3_CK	**2.755**	**0.0029**	**2.577**	**0.0050**	**0.0014**	**0.0027**
G3_CY	0.146	0.4421	−0.018	0.4929	0.5898	0.5898
G3_GH	−0.211	0.4165	−0.451	0.3260	0.5235	0.6603
G3_N1	−1.945	0.0259	−2.236	0.0127	0.9893	0.9964
G3_N2	0.435	0.3317	0.122	0.4514	0.7387	0.2899
G3_YJ	1.164	0.1223	0.923	0.1780	0.0770	0.0982
G4_BX	−1.439	0.0751	−2.067	0.0194	0.7793	0.8533
G4_HX	−0.109	0.4567	−0.624	0.2665	0.4159	0.6802
G4_LW	−0.069	0.4726	−0.201	0.4203	0.4816	0.4816
G4_WS	−0.194	0.4233	−0.643	0.2602	0.2450	0.2839
G4_YL	−0.740	0.2295	−0.965	0.1673	0.7378	0.7378
G5_DY	0.919	0.1790	0.557	0.2886	0.0844	0.1127
G5_JZ	**4.755**	**0.0000**	**4.642**	**0.0000**	**0.0000**	**0.0000**
G5_QH	−5.224	0.0000	−5.543	0.0000	1.0000	1.0000
G5_WQ	1.411	0.0791	1.071	0.1421	**0.0407**	0.0649
G5_XH	1.371	0.0851	1.206	0.1139	0.0523	0.0523
G5_YT	−0.485	0.3140	−0.512	0.3043	0.8125	0.8750
G6_HL	−0.230	0.4089	−0.844	0.1993	0.5218	0.8529
G6_JH	1.447	0.0739	0.977	0.1642	**0.0181**	0.0570
G6_KS	**4.213**	**0.0000**	**4.054**	**0.0000**	**0.0000**	**0.0000**
G6_LX	0.935	0.1749	0.648	0.2584	0.0978	0.2090
G6_QQ	−3.357	0.0004	−4.032	0.0000	0.9976	0.9994
G6_SY	−0.227	0.4102	−0.378	0.3528	0.4375	0.4375
G7_J1	**3.089**	**0.0010**	**2.903**	**0.0019**	**0.0001**	**0.0001**
G7_J2	−1.720	0.0428	−1.768	0.0385	1.0000	1.0000
G7_J3	0.979	0.1637	0.910	0.1815	0.0625	0.0625
G7_J4	−0.924	0.1777	−1.336	0.0907	0.5938	0.7392
G7_J5	1.356	0.0875	1.110	0.1335	**0.0327**	0.0523
G8_K1	−2.512	0.0060	−2.579	0.0050	1.0000	1.0000
G8_K2	−3.161	0.0008	−3.282	0.0005	1.0000	1.0000
G8_K3	−1.969	0.0245	−2.058	0.0198	0.9480	0.9710
G8_K4	−0.755	0.2253	−1.373	0.0848	0.6079	0.7848
G8_K5	**3.511**	**0.0002**	**3.299**	**0.0005**	**0.0004**	**0.0004**

SS: sum of squares; VC: variance component; PV: percentage of variation;

* *p* < 0.001; *F*_CT_: genetic diversity between two clusters; *F*_SC_: differentiation among populations within clusters; *F*_ST_: divergence among all populations. Significant for both tests are in bold.

**Table 5:** Locations and habitats of sampled wild soybean populations.

Geographical region	Population name	Location of sampling	Longitude	Latitude	Altitude (m)	Habitat
G1: SC	Population AF	Anfu county, Jiangxi province	27.388	114.602	85	Beside road
	Population JO	Jianou county, Fujian province	26.962	112.153	126	Beside river
	Population HY	Hengyang county, Hunan province	27.024	118.293	123	Beside river
	Population RY	Ruyuan county, Guangdong province	25.872	110.862	510	Beside road
	Population QZ	Quanzhou county, Guangxi province	24.919	113.136	722	Beside road

G2: MDYR	Population WC	Wuchang district, Hubei province	30.549	119.972	15	Beside road
	Population XU	Xuanwu district, Jiangsu province	31.314	117.128		Waste land
	Population DQ	Duqing county, Zhejiang province	32.370	113.400	15	Beside canal
	Population SC	Shucha county, Anhui province	30.521	114.395	45	Beside road
	Population TB	Tongbai county, Henan province	32.045	118.861	33	Beside road

G3: SWC	Population CK	Chengkou county, Chongqing	31.983	108.667	805	Valley
	Population YJ	Yinjiang county, Guizhou province	30.996	104.349	458	Valley,
	Population GH	Guanghan city, Sichuan province	28.000	108.406	458	Beside river
	Population CY	Chayu county, Xizang province	28.600	97.400	1685	Unknown
	Population NL1	Ninglang county, Yunnan province	27.455	100.758	2600	Beside filed
	Population NL2	Ninglang county, Yunnan province	27.340	100.954	2550	Beside filed

G4: NWC	Population BX	Bingxian county, Shaanxi province	35.040	108.077	835	Valley,
	Population HX	Huixian county, Gansu province	33.893	105.826	1126	Canal
	Population LW	Lingwu county, Ningxia province	38.146	106.326	1103	Canal
	Population WS	Wenshui county, Shanxi province	37.417	112.017	759	Beside canal
	Population YL	Yulin city, Shanxi province	38.281	109.738	1051	Along river

G5: NC	Population JZ	Jizhou county, Hebei province	37.574	118.524	23	Beside road
	Population DY	Dongying city, Shandong province	37.742	115.686	6	Beside ditches
	Population WQ	Wuqing district, Tianjing	39.808	119.432	−6	Beside ditches
	Population XH	Xuanhua county, Hebei province	39.449	117.249	601	Beside river
	Population QH	Qinghuangdao city, Hebei province	40.593	115.021	18	Beside river
	Population YT	Yantai city, Shandong province	37.485	121.453	10	Wasteland

G6: NEC	Population LX	Lanxi county, Heilongjiang province	41.893	123.411	139	Beside pond
	Population JH	Jiaohe county, Jinlin province	45.849	132.762	126	Beside river
	Population KS	Keshan county, Heilongjaing province	43.808	127.237	325	Aside field
	Population QQ	Qiqihaer city, Heilongjiang province	48.283	125.498	304	Beside river
	Population HL	Hulin city, Heilongjiang province	46.218	126.338	73	Beside filed
	Population SY	Shenyang, Liaoning province	47.341	123.940		wasteland

G7: Japan	Population J1	Kanagawa, Japan	34.960	137.160	12	Wet Land
	Population J2	Tokyo, Japan	34.828	135.770	35	Wet Land
	Population J3	Hirakata, Osaka, Japan	34.810	135.480	11	Wet Land
	Population J4	Okazaki, Japan	34.959	137.139	37	Wet Land
	Population J5	Kyushu University, Fukuoka, Japan	33.597	130.215		Unknown
	Population K1	Gangwon-do, South Korea	37.625	128.492	520	Wet Land
	Population K2	Gangwon-do, South, Korea	38.031	128.639	340	Wet Land
	Population K3	Incheon, South Korea	37.533	126.497	11	Wet Land
	Population K4	Yeongcheon-si city, Korea	36.113	128.982	102	Along road
	Population K5	Moonkyeong-si city, Korea	36.721	128.358	77	Along road

G1: SC, south China; G2: MDYR, Middle and lower reaches of Yangtze River; G3: SWC, southwestern China; G4: NWC, northwestern China; G5: NC, north China; G6: NEC, northeastern China.

**Table 6:** Characteristics of the 25 microsatellite loci for wild soybean. Forward (*F*) and reverse (*R*) primer sequences, repeat motifs, size range and linkage group are given.

Primer name	Primer sequence (5′ to 3′)	Repeat motif	Size range	linkage group
**gmcp1**	F:TCGATTCTATGCCCCTACTT R:AGACTCCCAAGTTTTCAGTCG	(T)12	124–126	TrnT/trnL
**gmcp3**	F:GCTTCAGAATTGTCCTATTTA R:ATCAAATAACGCCTCATCTA	(A)12CG(T)11	103–113	TrnT/trnL
**gmcp4**	F:TATCACTGTCAAGATTAAGAG R:CTTTTATATGTATGGCGCAAC	(A)11	127–136	atpB/rbcL
**RD19**	F:CTAAATATTACAAAATGGAATTCT R:ACCAATTCAAAAAATCGAATA	(A)14	149–151	rps19
**SOYCP**	F:CATAGATAGGTACCATCCTTTTT R:CGCCGTATGAAAGCAATAC	(T)13(G)10	90–98	trnM
**Satt126**	F:ATAAAACAAATTCGCTGATAT R:GCTTGGTAGCTGTAGGAA	(ATT)18	109–172	B2
**Satt135**	F:TTCCAATACCTCCCAACTAAC R:CACGGATTTTAAATCATTATTACAT	(ATT)19	141–204	D2
**Satt215**	F:GCGCCTTCTTCTGCTAAATCA R:CCCATTCAATTGAGATCCAAAATTAC	(ATT)11	114–221	J
**Satt216**	F:TACCCTTAATCACCGGACAA R:AGGGAACTAACACATTTAATCATCA	(ATT)20	137–251	D1b
**Satt221**	F:GCGGCAAACCATTATCTTCATT R:GCGATTGTACCACTAAAAACCATAG	(ATT)23	109–224	D1a
**Satt231**	F:GGCACGAATCAACATCAAAACTTC R:GCGTGTGCAAAATGTTCATCATCT	(ATT)32	160–328	E
**Satt233**	F:AAGCATACTCGTCGTAAC R:GCGGTGCAAAGATATTAGAAA	(ATT)16	169–238	A2
**Satt270**	F:TGTGATGCCCCTTTTCT R:GCGCAGTGCATGGTTTTCTCA	(ATT)16	183–249	I
**satt277**	F:GGTGGTGGCGGGTTACTATTACT R:CCACGCTTCAGTTGATTCTTACA	(ATT)40	128–255	C2
**satt288**	F:GCGGGGTGATTTAGTGTTTGACACCT R:GCGCTTATAATTAAGAGCAAAAGAAG	(ATT)17	195–273	G
**Satt294**	F:GCGCTCAGTGTGAAAGTTGTTTCTAT R:GCGGGTCAAATGCAAATTATTTTT	(ATT)23	237–303	C1
**Satt373**	F:TCCGCGAGATAAATTCGTAAAAT R:GGCCAGATACCCAAGTTGTACTTGT	(TAT)21	210–279	L
**Satt423**	F:TTCGCTTGGGTTCAGTTACTT R:GTTGGGGAATTAAAAAAATG	(ATT)19	225–351	F
**Satt463**	F:CTGCAAATTTGATGCACATGTGTCTA R:TTGGATCTCATATTCAAACTTTCAAG	(ATT)19	100–214	M
**satt509**	F:GCGCAAGTGGCCAGCTCATCTATT R:GCGCTACCGTGTGGTGGTGTGCTACCT	(ATT)30	119–242	B1
**Satt530**	F:CCAAGCGGGTGAAGAGGTTTTT R:CATGCATATTGACTTCATTATT	(ATT)12	201–279	N
**satt555**	F:GCGGTTGGCTTTGATGATGT R:TTACCGCATGTTCTTGGACTA	(ATT)13	234–312	K
**Satt568**	F:CGGACACCGGTCTACTAGGAAAGTAA R:GCGGAATAATCCAATTCAATTTA	(ATT)17	212–275	H
**satt572**	F:GCGGAGCATGTAAATCCAGCCTATTGA R:GCGGGCTAACTTATGTTACTAAACAAT	(ATT)14	130–241	A1
**satt581**	F:CCAAAGCTGAGCAGCTGATAACT R:CCCTCACTCCTAGATTATTTGTTGT	(ATT)11	130–196	O
